# Epidermoid cyst arising from an intrapancreatic accessory spleen: A case report and review of the literature

**DOI:** 10.3892/ol.2012.1061

**Published:** 2012-12-04

**Authors:** RAN HONG, NAMKYU CHOI, KYUNGHOON SUN, SHARON LIM, YUNJU HAN

**Affiliations:** 1 Departments of Pathology, College of Medicine, Chosun University, Gwangju 501-759, Republic of Korea; 2 Surgery, College of Medicine, Chosun University, Gwangju 501-759, Republic of Korea; 3 Emergency Medicine, College of Medicine, Chosun University, Gwangju 501-759, Republic of Korea

**Keywords:** accessory spleen, pancreas, cyst

## Abstract

We describe an epidermoid cyst arising from an accessory spleen of the pancreas. A 56-year-old female with iron deficiency anemia presented with dizziness. During the clinical workup, a 2×4 cm-sized mass was incidentally detected in the tail of the pancreas in a computed tomography (CT) scan. Under a clinical diagnosis of pancreatic cystic malignant tumor, a distal pancreatectomy was carried out. Grossly, the lesion was composed of a solid and cystic portion. Microscopic analysis revealed that the solid portion was an intrapancreatic accessory spleen and the cystic portion was an epidermoid cyst. An epidermoid cyst in an intrapancreatic accessory spleen is extremely rare and hence difficult to diagnose pre-operatively. Taking into account the possibility of such a cyst in the differential diagnosis of intrapancreatic cystic lesion is recommended.

## Introduction

The occurrence of an accessory spleen is not rare; it affects approximately 10% of the general population, and 16% of all cases are intrapancreatic (1), However, the development of an epidermoid cyst of intrapancreatic accessory spleen (IPAS) is not common, with 30 cases (2–29) described in the literature since Davidson *et al*(2) described the first case of epidermoid cyst of IPAS. Due to the difficulty in differentiating the lesion from a cystic neoplasm of the pancreas by an imaging study (4), the majority have been diagnosed following surgical resection, with the exception of one case by Itano *et al*(5). Pre-operative diagnosis was mainly cystic neoplasm of the pancreas. Herein, we report a case of 54-year-old female with an epidermoid cyst of an IPAS and review the literature.

The study was approved by the ethics committee of Chosun University Hospital, Gwangju, Korea (IRB No.: CHOSUN 2012-10-007). The committee approved the waiver of patient consent for these cases.

## Case report

**Figure 1.**
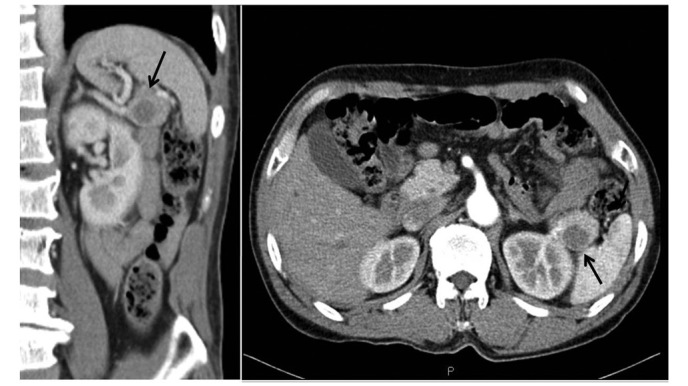
Radiological examination. Enhanced abdominal computed tomography (CT) reveals a clearly defined, 2.3 cm-sized nodule in the pancreatic tail (arrow).

**Figure 2.**
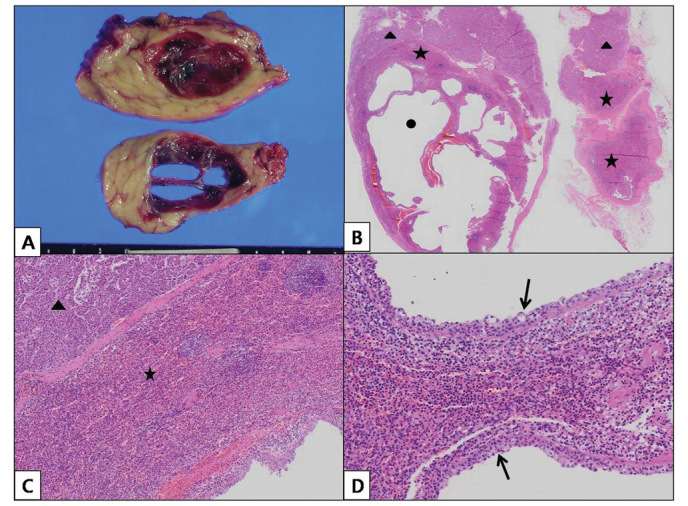
Histopathological examination. (A) Grossly, the cut surface of the pancreas exhibits a multilocular cystic mass measuring 2.0×1.5 cm. (B,C) Microscopic analysis reveals a multilocular cyst (circle) surrounded by accessory splenic tissue (star) in the pancreas parenchyma (triangle). (D) The cyst is lined by multilayered cuboidal epithelium (arrow).

A 54-year-old female with iron deficiency anemia was admitted to hospital complaining of dizziness and abdominal discomfort. During the clinical workup, a 2.3 cm-radiological-sized cystic mass was detected in the tail of the pancreas by abdominal computed tomography (CT; Fig. 1). Distal pancreatectomy was performed upon clinical diagnosis of pancreatic cancer. Grossly, the surgical specimen showed a well-demarcated multilocular cystic mass within the pancreatic parenchyma, measuring 2.0×1.5 cm (histoligcal size) and containing dark serosanginous fluid. Microscopic investigation revealed that the majority of the epithelial lining was comprised of multilayered cuboidal epithelium with focal denudation. However, no atypical or malignant changes were observed (Fig. 2). Immunohistochemical staining demonstrated that the epithelial lining was reactive for cytokeratin (CK) and CK7. The cystic wall demonstrated histologically normal splenic pulp tissue, which was surrounded by a hyalinized fibrous band. The final pathologic diagnosis was an epidermoid cyst arising from an IPAS. The six-month post-operative course was uneventful.

## Discussion

Approximately 16% of accessory spleens occur in or around the tail of the pancreas (1). An epidermoid cyst in an IPAS is extremely rare and was first described in 1980 by Davidson *et al*(2). Following this, 30 cases of epidermoid cyst of IPAS have been described in the literature. Table I summarizes the 31 cases of epidermoid cyst in an IPAS, including the case we describe here. The cases involved 15 males and 16 females, with ages ranging from 12–70 years (mean, 46 years). All cysts were located in the pancreatic tail. While 16 patients were asymptomatic, various symptoms were observed in 14 patients, including weight loss, nausea, vomiting, abdominal pain and discomfort, back pain, epigastric pain and fever. Histological analysis revealed that the cysts were solitary or multilocular, lined with keratinized or non-keratinized stratified squamous epithelium or cuboidal epithelium, and in some cases exhibiting mixed-form epithelium.

An elevation of serum CA 19-9 level was observed in 10 cases, hence the difficulty in pre-operatively differentiating between an epidermoid cyst in an IPAS and pancreatic malignancy during clinical analysis. Higaki *et al*(9) revealed that the serum CA 19-9 level markedly decreased to within the normal range following surgery in a patient diagnosed with an epidermoid cyst in an IPAS, suggesting that the serum CA 19-9 originated in the epidermoid cyst in an IPAS.

The histogenesis of an epidermoid cyst in an IPAS may be identical to that of a splenic epidermoid cyst (23). There are three hypotheses concerning the histogenesis of an epidermoid cyst in an IPAS (10). Firstly, the cyst may originate from mesothelial inclusion with subsequent squamous metaplasa (30). Secondly, teratomatous derivation or an inclusion of fetal squamous epithelium may cause cystic change (31). Thirdly, a derivation from the pancreatic duct may protrude into the accessory spleen (10). In a case described by Kadota *et al*(23), there were pancreatic ducts in the fibrous tissue surrounding the accessory spleen tissue, and the squamous and cuboidal epithelia indicated a transitional appearance from one form to the other. Additionally, immunohistochemical analysis demonstrated that the staining results of the cystic epithelial lining were identical to those of the pancreatic duct. These results support the third hypothesis.

A pre-operative imaging diagnosis of an epidermoid cyst in an IPAS is extremely difficult. Notably, a diagnosis of abdominal CT in the present case was also pancreatic tail cancer. As there are no characteristic features to define the lesion on radiology, it is not possible to entirely differentiate the cystic pancreatic malignancy prior to surgery and histopathological examination (28).

In conclusion, an epidermoid cyst in an IPAS is an extremely rare disease entity, and radiographic and clinical results (including CA 19-9 elevation) are similar to those of other cystic pancreatic neoplasms. As a result, the possibilty of such a cystic lesion should be considered in the differential diagnosis of a pancreatic cystic lesion.

## Figures and Tables

**Table I.** Summary of the 31 cases of epidermoid cyst arising in intrapancreatic accessory spleen (IPAS), including the present case.

No	First author (Ref.)	Year	Age/Gender	Site	Symptoms	CA 19-9	Size (cm)	Surgery	Epi. lining
1	Davidson (2)	1980	40/M	Tail	WL, N	NR	5.5	DP&S	-
2	Hanada (3)	1981	51/M	Tail	AP	NR	-	DP	-
3	Jibu (4)	1987	37/M	Tail	-	-	4.0	-	-
4	Morohoshi (5)	1991	32/M	Tail	AP	WNL	6.0×5.0	RC	SSE
5	Nakae(6)	1991	37/F	Tail	AP	NR		SPDP	
6	Tang (7)	1994	38/M	Tail	ASx.	NR	1.4	DP	SSE
7	Furukawa (8)	1998	45/M	Tail	ASx.	WNL	2.0	DP	SSE
8	Higaki (9)	1998	46/F	Tail	Back pain	201	3.0×3.0	DP&S	SSE
9	Tateyama (10)	1998	67/F	Tail	AP	201	3.0	DP	SSE
10	Sasou (11)	1999	49/F	Tail	ASx.	WNL	4.3×2.6	DP	NSSE
11	Tsutsumi (12)	2000	51/M	Tail	ASx.	WNL	2.5	DP	NSSE
12	Choi (13)	2000	54/F	Tail	EP, N, V, WL	NR	15.0×11.0	E&S	KSSE
13	Horibe (14)	2001	48/M	Tail	ASx.	53	2.0×1.0	DP	SSE
14	Sonomura (15)	2002	45/F	Tail	EP	159	3.5	DP	SSE
15	Yokomizo (16)	2002	38/M	Tail	ASx.	410	2.7	DP	NSSE
16	Fink (17)	2002	12/F	Tail	Fever	NR	10.0	RC	NSSE
17	Kanazawa (18)	2004	58/F	Tail	ASx.	62	2.5	SPDP	SSE
18	Ru (19)	2007	41/M	Tail	ASx.	NR	2,5	DP	NSSE
19	Itano (20)	2008	40/M	Tail	ASx.	WNL	3.0	DP	SSE
20	Servais (21)	2008	52/F	Tail	ASx.	NR	11.5×10.5×8.5	DP	CCE
21	Gleeson (22)	2008	32/F	Tail	AP	NR	1.5×1.2	DP&S	SSE
22	Kadota (23)	2009	57/F	Tail	ASx.	WNL	6.0×5.0×4.0	DP	NSSE, CE
23	Kadota (23)	2009	70/F	Tail	ASx.	48	1.7×1.0×0.8	DP	NSSE, CE
24	Kadota (23)	2009	37/M	Tail	ASx.	647	10×7.0×7.0	SPDP	KSSE, CE
25	Zhang (24)	2009	26/F	Tail	ASx.	WN	2.5×2.5	SPDP	SSE
26	Itano (25)	2010	67/M	Tail	EP, WL	WNL	3.0	LADP	SSE
27	Yamanishi (26)	2011	55/F	Tail	ASx.	90	2.5×1.5	DP	SSE
28	Iwasaki (27)	2011	36/F	Tail	EP, WL	79	3.4×1.9	LDP	SSE
29	Horn (28)	2011	62/M	Tail	AP	NR	4.8×3.7×1.9	CR	KSSE
30	Khashab (29)	2011	49/F	Tail	AP	NR	2.3	LADP	SSE
31	Present case	2012	56/M	Tail	AP.	WNL	2.0×1.5	SPDP	CE

M, male; F, female; Epi. lining, epithelial lining; EP, epigastric pain; N, nausea; V, vomiting; WL, weight loss; ASx., asymptomatic; CA 19-9, carbohydrate antigen 19-9 (IU/ml); DP, distal pancreatectomy; LADP, laparoscopic-assisted distal pancreatectomy; LDP, laparoscopic distal pancreatectomy; NR, not reported; RC, removal of the cyst; SPDP, spleen-preserving distal pancreatectomy; ES, explorolaparotomy; S, splenectomy; NSSE, non-keratinizing stratified squamous epithelium; CE, cuboidal epithelium; KSSE, keratinizing stratified squamous epithelium; SSE, stratified squamous epithelium; CCE, columnar and cuboidal epithelium.
